# Supplementary material for: Behavioural relevance of spontaneous, transient brain network interactions in fMRI
Source: Neuroimage. 2021 Apr 1;229:117713. doi: 10.1016/j.neuroimage.2020.117713 (PMC7994296; doi:10.1016/j.neuroimage.2020.117713)
Supplement: Supplementary file 1 [file mmc1.docx]

**Supplemental Information**


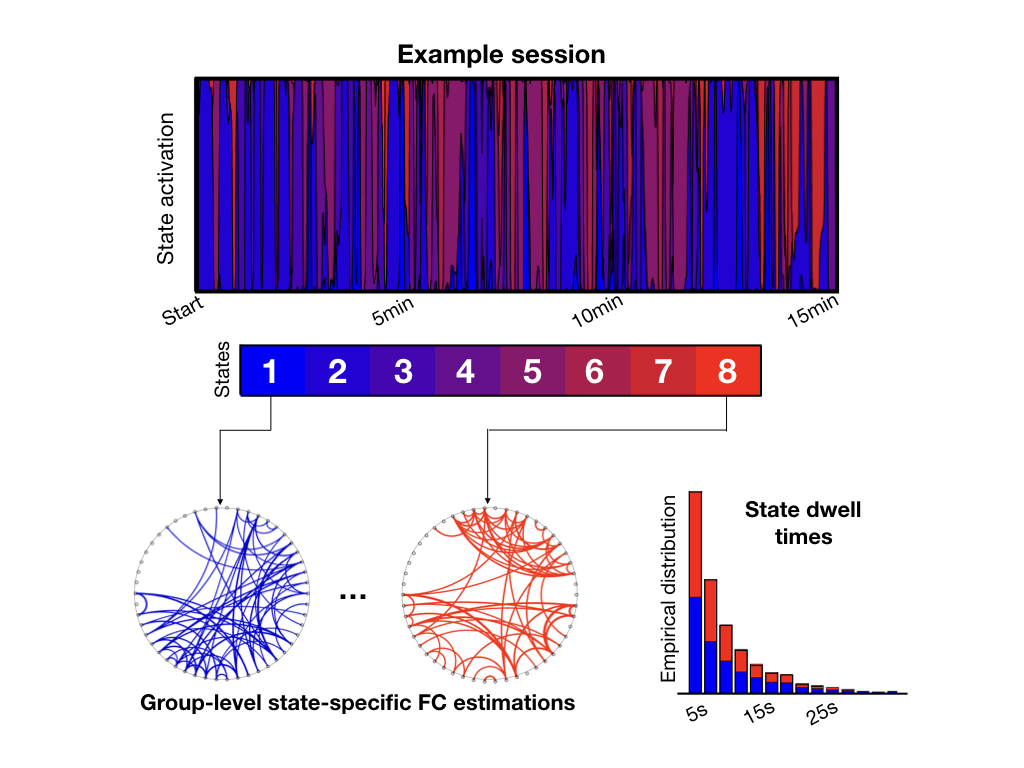


**Fig SI-1**. Illustration of the HMM description of time-varying FC, for an example session; states are represented as connectivity matrices. On the bottom right, depiction of the distribution of state dwell times for two of the states, at the group level.

**
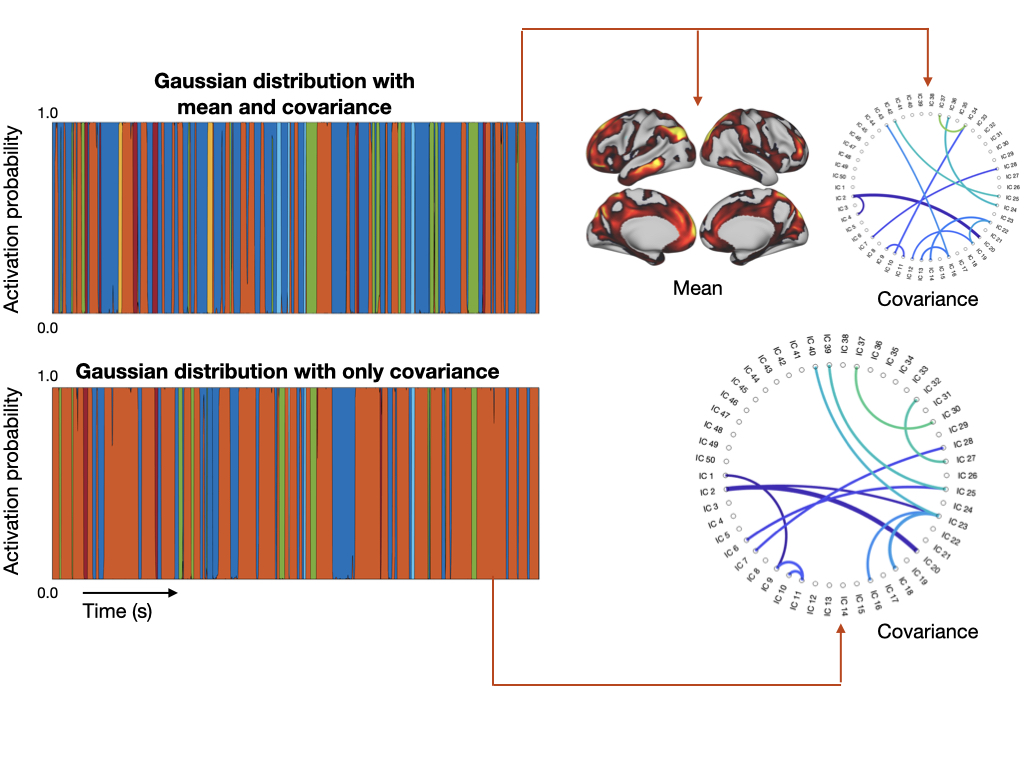
**

**Fig SI-2**. Example of the HMM run on just one subject, for two varieties of the HMM: The standard HMM with states defined by Gaussian distribution with mean and covariance (Vidaurre et al., 2017), and the FC-HMM used here which only uses covariance. On the left, state time courses for one session; on the right, observation model for one state. The random seeds were equal for both runs.


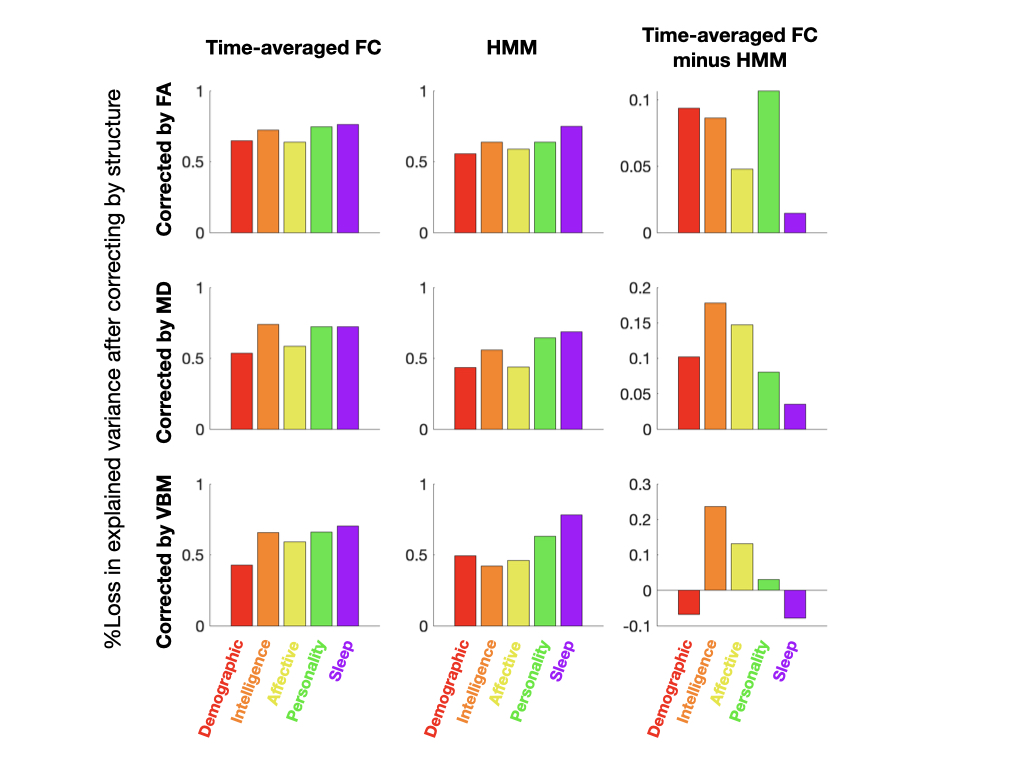


**Fig SI-3**. Loss in explained variance of the deconfounded predictions with respect to the non-deconfounded predictions, expressed as a percentage. For the right panels on the right, positive values indicate that the time-average FC descriptor lost more explained variance than the time-varying FC descriptor after correcting for the structure, and negative values indicate contrariwise.

**
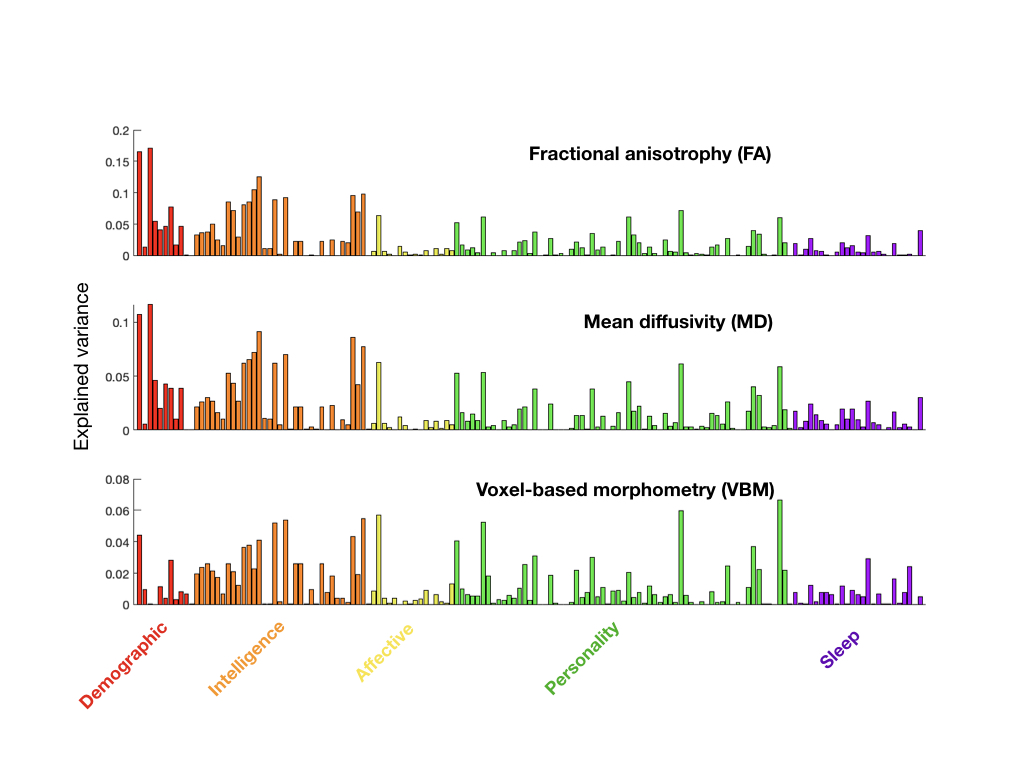
**

**Fig SI-4**. Explained variance *r^2^* (in terms of squared Pearson’s correlation) of each of behavioural trait by the structural information. Traits are coloured according to five different behavioural groups: demographics, intelligence, affective, personality and sleep.


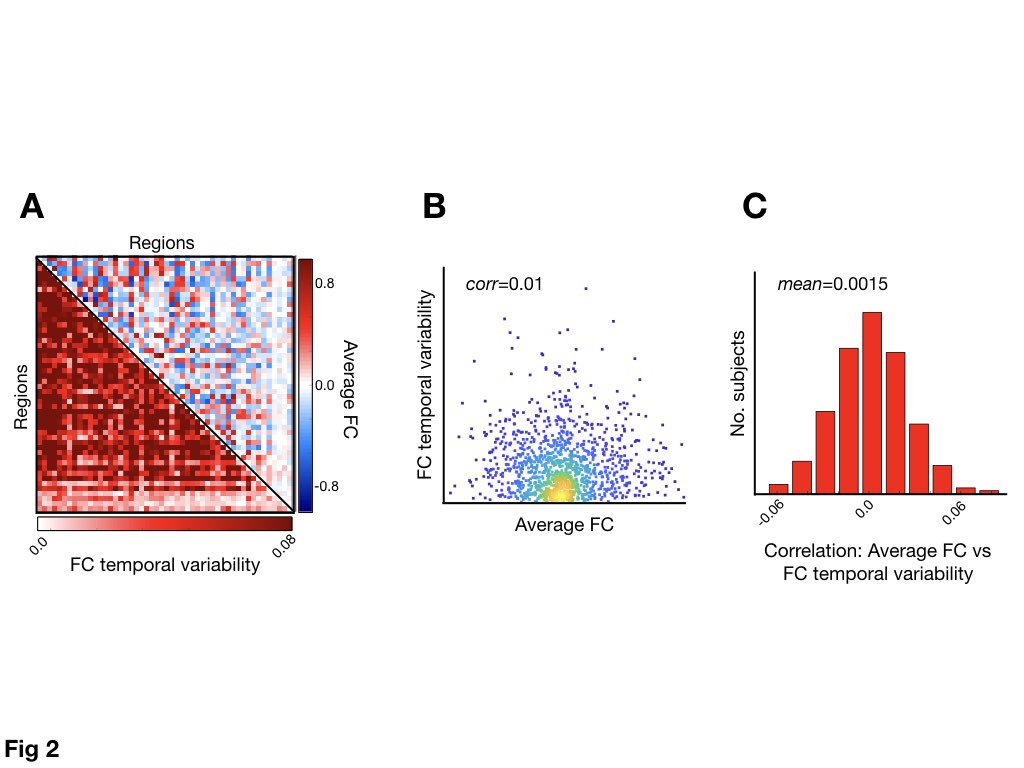


**Fig SI-5.** In order for FC temporal variability and time-averaged FC to explain distinct aspects of behaviour, these representations must contain non-shared elements of information of brain function. This amounts to showing that there is unique, subject-specific behaviourally-relevant information in the time-varying FC, which is not contained in the time-averaged FC. To do this, we computed the time-averaged FC for each subject and compared it with a measure of FC temporal variability for each subject (see Methods). This figure shows that the amount of time-averaged FC for any pair of regions is unrelated to amount of FC temporal variability for such a pair: (**A)** an example for one subject, where the upper triangular matrix represents time-averaged FC and the lower triangular matrix represents FC temporal variability; **(B)** the relation between time-averaged FC and FC temporal variability for that same subject as a scatter plot, where each dot corresponds to a pair of regions. Although the null hypothesis cannot be proved in this way, we note that the correlation between these measures is 0.01 and non-significant; **(C)** histogram of correlations between time-averaged FC and FC temporal variability across subjects; the mean correlation is 0.0015 and is non-significantly positive.

| **Trait group** | **Traits** |
| --- | --- |
| Demographic | Age, handedness, race, ethnicity, SSAGA_Employ, SSAGA_Income, SSAGA_Educ, SSAGA_InSchool, SSAGA_Rlshp, SSAGA_MOBorn |
| Intelligence | MMSE_Score, PicSeq_Unadj, PicSeq_AgeAdj, CardSort_Unadj, CardSort_AgeAdj, Flanker_Unadj, Flanker_AgeAdj, PMAT24_A_CR, PMAT24_A_SI, PMAT24_A_RTCR, ReadEng_Unadj, ReadEng_AgeAdj, PicVocab_Unadj, PicVocab_AgeAdj, ProcSpeed_Unadj, ProcSpeed_AgeAdj, VSPLOT_TC, VSPLOT_CRTE, VSPLOT_OFF, SCPT_TP, SCPT_TN, SCPT_FP, SCPT_FN, SCPT_TPRT, SCPT_SEN, SCPT_SPEC, SCPT_LRNR, IWRD_TOT, IWRD_RTC, ListSort_Unadj, ListSort_AgeAdj |
| Affective | AngAffect_Unadj, AngHostil_Unadj, AngAggr_Unadj, FearAffect_Unadj, FearSomat_Unadj, Sadness_Unadj, LifeSatisf_Unadj, MeanPurp_Unadj, PosAffect_Unadj, Friendship_Unadj, Loneliness_Unadj, PercHostil_Unadj, PercReject_Unadj, EmotSupp_Unadj, InstruSupp_Unadj, PercStress_Unadj, SelfEff_Unadj |
| Personality | NEOFAC_A, NEOFAC_O, NEOFAC_C, NEOFAC_N, NEOFAC_E, NEORAW_01, NEORAW_02, NEORAW_03, NEORAW_04, NEORAW_05, NEORAW_06, NEORAW_07, NEORAW_08, NEORAW_09, NEORAW_10, NEORAW_11, NEORAW_12, NEORAW_13, NEORAW_14, NEORAW_15, NEORAW_16, NEORAW_17, NEORAW_18, NEORAW_19, NEORAW_20, NEORAW_21, NEORAW_22, NEORAW_23, NEORAW_24, NEORAW_25, NEORAW_26, NEORAW_27, NEORAW_28, NEORAW_29, NEORAW_30, NEORAW_31, NEORAW_32, NEORAW_33, NEORAW_34, NEORAW_35, NEORAW_36, NEORAW_37, NEORAW_38, NEORAW_39, NEORAW_40, NEORAW_41, NEORAW_42, NEORAW_43, NEORAW_44, NEORAW_45, NEORAW_46, NEORAW_47, NEORAW_48, NEORAW_49, NEORAW_50, NEORAW_51, NEORAW_52, NEORAW_53, NEORAW_54, NEORAW_55, NEORAW_56, NEORAW_57, NEORAW_58, NEORAW_59, NEORAW_60' |
| Sleep | PSQI_Score, PSQI_Comp1, PSQI_Comp2, PSQI_Comp3, PSQI_Comp4, PSQI_Comp5, PSQI_Comp6, PSQI_Comp7, PSQI_Min2Asleep, PSQI_AmtSleep, PSQI_Latency30Min, PSQI_WakeUp, PSQI_Bathroom, PSQI_Breathe, PSQI_Snore, PSQI_TooCold, PSQI_TooHot, PSQI_BadDream, PSQI_Pain, PSQI_Other, PSQI_Quality, PSQI_SleepMeds, PSQI_DayStayAwake, PSQI_DayEnthusiasm, PSQI_BedPtnrRmate |

**Table SI-1**. List of behavioural and anatomical traits per group.
